# Adult urinary bladder tumors with rabdomyosarcomatous differentiation: Clinical, pathological and immunohistochemical studies

**DOI:** 10.1186/1746-1596-6-66

**Published:** 2011-07-15

**Authors:** Zhanyong Bing, Paul J Zhang

**Affiliations:** 1 Department of Pathology and Laboratory Medicine, Hospital of the University of Pennsylvania, Philadelphia, PA, USA

## Abstract

Adult rhabdomyosarcoma (RMS) in the urinary bladder is rare, and is the subject of case reports and small series. It consists of sheets of small round blue cells with high nuclear cytoplasmic ratio, brisk mitosis and apoptosis. In this study, we reported one case of pure rhabdomyosarcoma and two cases of urothelial carcinomas with extensive rhabdomyosarcomatous differentiation. In addition, their immunohistochemical profile was compared to that of small cell carcinoma of the bladder. Our study showed that sufficient sampling was critical for the diagnosis of urothelial carcinoma with extensive rhabdomyosarcomatous differentiation. As adult RMS in the bladder and urothelial carcinoma with rhabdomyosarcomatous differentiation shared morphological features with small cell carcinoma of the bladder, appropriate immunohistochemical stains were necessary in the differential diagnosis. We showed both rhabdomyosarcoma and rhabdomyosarcomatous areas of the urothelial carcinoma were positive for myogenin, negative for cytokeratin and chromogranin stains. In contrast, small cell carcinoma was positive for cytokeratin, and 7 out of 9 cases were also positive for chromogranin. Both rhabdomyosarcoma and small cell carcinoma could be positive for synaptophysin, a potential pitfall to avoid. In addition, all of the tumors with rhabdomyosarcomatous differentiation were negative for FKHR rearrangement.

## Introduction

Rhabdomyosarcoma occurs much more commonly in children than in adults in genitourinary tract, in which more than 90% is embryonal, mostly botryoid type. Adult rhabdomyosarcoma is extremely rare [1-7]. Because of its rarity in adults, proper diagnosis and classification can be a challenge. To make this matter more complicated, rhabdomyoblastic differentiation is one of commonly seen heterologous elements in sarcomatoid carcinoma of urinary bladder [7]. Adult rhabdomyosarcoma of urinary bladder can have morphologic features overlapping with small cell carcinoma and poorly differentiated urothelial carcinoma with rhabdomyoblastic differentiation [7]. In this report, we reported three cases of primary rhabdomyosarcomatous tumor of the urinary bladder, two of them were diagnosed as carcinoma with extensive rhabdomyosarcomatous differentiation based on very minor carcinomatous elements identified and one as rhabdomyosarcoma without carcinomatous elements identified in the tissue examined. In addition, we compared the histologic and immunohistochemical features of these urinary bladder tumors with rhabdomyosarcomatous differentiation to that of well characterized bladder small cell carcinoma.

## Material and methods

The database of the Department of Pathology and Laboratory Medicine of the University of Pennsylvania was searched for bladder rhabdomyosarcoma, carcinoma with rhabdomyosarcomatous differentiation and small cell carcinoma for the period 1987-2010. One case of rhabdomyosarcoma and two cases of poorly differentiated urothelial carcinoma with extensive rhabdomyosarcomatous differentiation were identified, and the medical charts were reviewed and reported. In addition, 8 cases of pure bladder small cell carcinoma and 5 cases of poorly differentiated urothelial carcinoma with areas of small cell carcinoma were retrieved for the study. All specimens were fixed in a 10% neutral-buffered formalin solution and processed routinely. Histologic sections were reviewed and representative blocks of each case were selected for immunohistochemical study. This research was approved by the University of Pennsylvania Institutional Review Board.

### Immunohistochemistry

Immunohistochemical stain was performed on formalin-fixed, paraffin-embedded 4-um tissue sections with the avidin-biotin immunoperoxidase complex method (LSAB2 system, Dako Corporation, Carpinteria, Calif) with diaminobenzedine as the chromogen and hematoxylin as the nuclear counterstain. Information about the antibodies used was summarized in Table 1. Antigens retrieval was done by incubating the tissue sections in a Black and Decker Vegetable Steamer for 20 minutes in Target Retrieval Solution (Dako) preheated to 99°C. The negative control was performed by substituting the primary antibody with nonimmune mouse or rabbit serum. Approximate positive controls were used.

**Table 1:** Antibodies

Antibody	Type	Manufacturer	Dilution
**Myogenin**	Mouse monoclonal	Dako	1:200

**AE1/3**	Mouse monoclonal	Novacastra Laboratories	1:400

**Pan-CK**	Mouse monoclonal	Novacastra Laboratories	1:50

**Chromogranin**	Rabbit polyclonal	Zymed/Invitrogen	1:100

**Synaptophysin**	Rabbit polyclonal	Zymed/Invitrogen	1:50

### FISH

FISH assay for translocation involving EWS and FHKR was performed on paraffin sections using Vysis breakapart probes (Vysis) according to the manufacture's procedure guidelines.

### Case histories and pathology

Primary bladder tumors with rhabdomyosarcomatous differentiation: tumor with pure rhabdomyoblastic differentiation a.k.a rhabdomyosarcoma (case 1) versus tumor with minor carcinomatous element, a.k.a rhabdomyosarcomatous carcinoma (cases 2 and 3).

**Figure 1:**
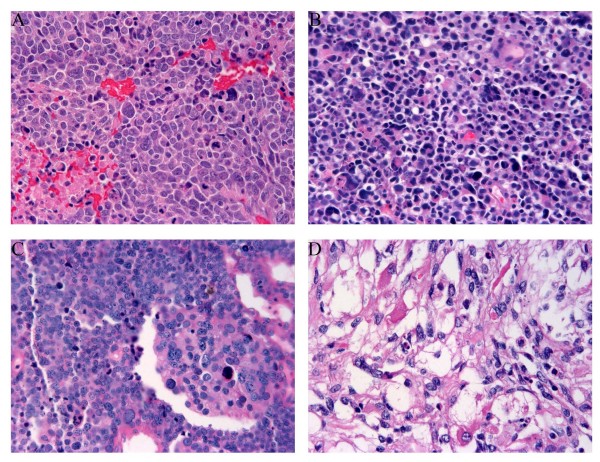
**Morphology of primary bladder rhabdomyosarcoma and bladder tumor with extensive rhabdomyosarcomatous and focal epithelial differentiation, H&E, 400x**. A. Primary rhabdomyosarcoma with anaplasia. B, C, D. Bladder tumor with extensive rhabdomyosarcomatous differentiation and focal epithelial differentiation showing anaplasia 400x (B), focal epithelial differentiation (C), rhabdomyoblastic differentiation (D).

The clinical and histological features were summarized in table 2 and 3. All are adult patient (one male and two female). All presented with hematuria, two underwent cystectomy and the third one underwent transurethral resection of bladder tumor (TURBT) twice. The tumors were exophytic and measured 3.2 cm in case 1 and 25 cm in case 2 in the cystectomy specimens. In case 3 the second TURBT was performed in this institution and consisted of tumoral tissue in fragments in three parts measuring 5.5 × 5.5 × 1.0 cm, 2.5 × 2.5 × 0.5 cm and 3.0 × 3.5 × 0.7 cm in aggregates respectively, with largest fragments measuring 1.6 cm, 1.7 cm and 1.7 cm respectively. All three tumors showed areas of small round cell morphology with brisk mitosis and uniformly small nuclei with no prominent nucleoli (Figure 1A, B). The pure rhabdomyosarcoma (Case 1) showed no apparent rhabdomyoblasts, while case 2 showed extensive rhabdomyoblastic features and case 3 showed focal rhabdomyoblastic features (Figure 1D). In addition to prominent small round cell feature, all of the three cases exhibited scattered marked nuclear anaplasia (Figure 1A, B) and pleomorphism and patchy coagulative necrosis. No distinct alveolar pattern is seen. No carcinomatous element was identified in pure rhabdomyosarcoma in 25 sections of the 3.2 cm tumor, and the carcinomatous elements were very focal in other two cases (Figure 1C). In case 2, rare neoplastic epithelial cells were seen in pagetoid spread in the overlying urothelium in extensive sampling of this large tumor in one out of 46 sections. In case 3, very focal invasive urothelial carcinoma component was identified only in the initial TURBT specimen and not in the second TURBT specimens despite the fact that all of the tissue was submitted for histologic evaluation. Tumors in case 1 and 2 showed deep invasion into perivesicular tissue with lymph node metastasis.

**Table 2:** Clinical features

**Case No**.	Age (years)/Gender	Clinical presentation	Treatment
**1**	61/Female	Hematuria	Cystectomy

**2**	58/Male	Hematuria	Cystectomy

**3**	57/Female	Hematuria	TURBT

**Table 3:** Histologic features

Case	Size (cm)	Carcinoma Component	Mitosis (HPF)	Necrosis	Anaplasia	Rhabdomyoblasts	Depth of invasion	Lymph node Metastasis
**1**	3.2	No	17	Yes	Yes	No	perivesicular tissue	4/35

**2**	25	Yes	3	Yes	Yes	Yes (diffuse)	PVT	1/1

**3**	4 (ultrasound)	Yes (very focal)	9	Yes	Yes	Yes	At least detrusor muscle	N/A

Bladder tumors with small cell carcinoma component: Pure small cell carcinoma and poorly differentiated urothelial carcinoma with small cell carcinoma components.

**Figure 2:**
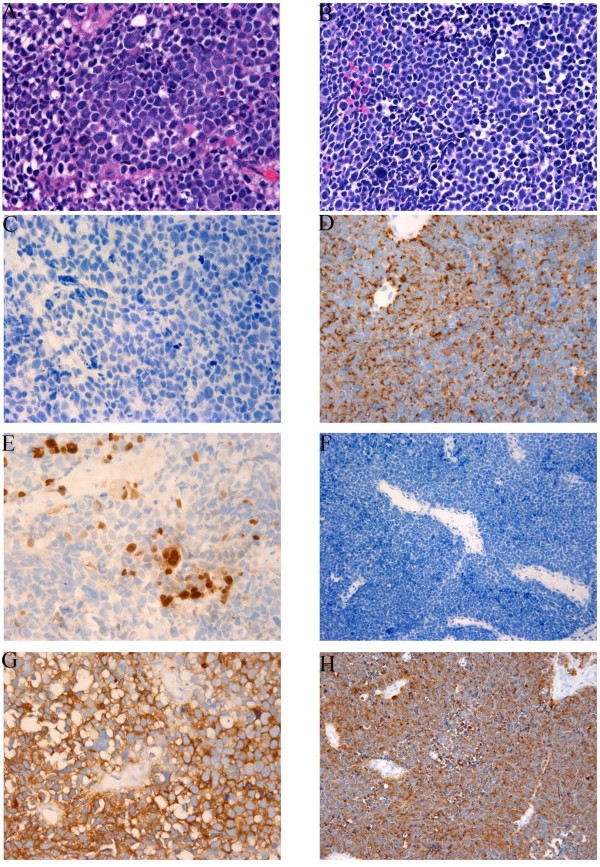
**Immunohistochemical profiles of primary urinary bladder rhabdomyosarcoma and small cell carcinoma (400x)**. A, C, E, G, primary urinary bladder rhabdomyosarcoma. A, H&E; C. Cytokeratin A/E1/3; E. Myogenin; G. Synaptophysin; B, D, F, H, small cell carcinoma of the urinary bladder. B. H&E; D. Cytokeratin AE1/3; F. Myogenin; H. Synaptophysin.

There is no morphologic difference in the small cell carcinoma components between pure small cell carcinoma and poorly differentiated urothelial carcinoma with small cell carcinoma components. The tumors were composed of loosely arranged sheets and nests of small round blue cells with brisk mitosis and salt pepper chromotins (Figure 2B). Nuclear anaplasia was common. No rhabdomyoblastic differentiation is identified. The only difference was that areas of poorly differentiated conventional urothelial carcinoma were seen in the latter group.

### Immunohistochemical study

Immunohistochemical studies with adequate controls were performed and the results were summarized in table 4. As shown in the table, the rhabdomyosarcomatous areas of all of three rhabdomyosarcomatous tumors of the bladder showed positivity for myogenin and desmin. Both of the pure rhabdomyosarcoma or rhabdomyosarcomatous carcinoma (carcinoma with extensive rhabdomyosarcomatous differentiation) showed similar extensive myogenin positivity (Figure 2E). No cytokeratin (Figure 2C) or chromogranin reactivity was detected in the rhabdomyosarcomatous areas of these tumors. Cytokeratin reactivity was only restricted to the rare pagetoid carcinomatous cells in case 2 and focal urothelial carcinoma in the case 3. Interestingly, Two out of three tumors (case 1 and 2) were also diffusely positive for synaptophysin (Figure 2G), but negative for chromogranin. Six out of 8 pure small cell carcinoma of the bladder and 3 out of 5 small cell carcinoma components in poorly differentiated urothelial carcinoma were evaluated for cytokeratin, chromogranin and synaptophysin reactivity. All cases tested were positive for cytokeratin (Figure 2D). All but 2 were positive for chromogranin. 7 cases of small cell carcinoma and 2 case of small cell carcinoma components of urothelial carcinoma were also stained for synaptophysin and all were positive (Figure 2H). All of 13 cases of the small cell carcinoma component from the pure small cell carcinoma and urothelial carcinoma with small cell carcinoma components were negative for myogenin (Figure 2F).

**Table 4:** Immunohistochemical study of Rhabdomyosarcomatous tumors and small cell carcinoma

Cases	Number of cases	Myogenin	Cytokeratin	Synaptophysin	Chromogranin	FISH in RMS
RMS*	1	Positive	Negative	Positive	Negative	negative

**RMS in ca^**	2	Positive in RMS	Negative in RMS; positive in ca	Positive in RMS in 1 out of 2 cases	Negative	negative

**Small cell carcinoma**	13	Negative	Positive (9/9)	Positive(9/9)	Positive in 7/9	N/A

FISH DNA breakapart signal analysis for detection of translocation associated with EWS and FHKR.

*RMS: Rhabdomyosarcoma or rhabdomyosarcomatous component in appropriate setting; ^ca: Carcinoma.

### FISH assays

No EWS and FHKR breakapart signals were detected in any of three rhabdomyosarcomatous areas of the three bladder tumors.

## Discussion

RMS in the urinary bladder has been well documented in children with the majority being embryonal, betryoid type [8,9]. RMS in adult urinary bladder is rare, with only scattered case reports or small series [2,6,7,10-15]. In the reported cases, the tumor usually occurs in older patients with the average age of 63+/-13 years. There is a predilection for men with a male to female ration roughly 2:1.

RMS in adult is composed of small round blue cells with high nuclear cytoplasm ratio, brisk mitosis and frequent apoptosis. Frequently the tumors show nuclear anaplasia, with random large anaplastic cells scattered in the tumor, similar to the nuclear anaplasia seen in the Wilm's tumor without the requirement of tripolar atypical mitosis [16]. RMS in adult urinary bladders has been reported to be alveolar [7,14,17], embryonal [11-13,17], pleomorphic [2,6] type or unspecified [10].

Rhabdomyoblastic morphology may not be present in the tumor; therefore it may be confused with other primitive tumors, especially with small cell carcinoma. On the other hand, variable aberrant rhabdomyoblastic differentiation could be seen in poorly differentiated urothelial carcinoma. Typically, differential diagnosis of RMS in adults includes sarcomatoid urothelial carcinoma with extensive rhabdomyosarcomatous differentiation and other tumors with small round cell morphology including small cell carcinoma, plasmacytoid urothelial carcinoma, primitive neuroectodermal tumor and lymphoma. Immunohistochemical analysis has an important role in the differential diagnosis of these tumors. Desmin and myogenin immunoreactivity can be used to differentiate rhabdomyosarcoma from other round cell tumors without rhabdomyoblastic differentiation but not sarcomatoid carcinoma with rhabdomyoblastic differentiation. In practice, urothelial carcinoma with extensive rhabdomyosarcomatous differentiation can only be differentiated from de novo rhabdomyosarcoma based on finding of any carcinomatous (neoplastic epithelial) element and/or unequivocal cytokeratin immunoreactivity in tumor cells. Therefore, sufficient sampling and mapping of tumor to look for any carcinomatous element, in-situ or invasive, are critical for making the distinction between these two entities. The diagnostic difficulties are reflected in two of our cases. In case 2, the tumor is very large (25 cm) and only very small focus showing carcinomatous differentiation. In case 3, small focus of urothelial carcinoma was only identified in the initial TURBT specimen. In the subsequent TURBT, no carcinomatous component was identified albeit the whole specimen was entirely submitted in 15 cassettes for histologic evaluation. One can conceive that when the carcinomatous element is very small and/or dominated or replaced by rhabdomyosarcomatous element, the minor carcinomatous element might not be morphologically detectable easily in routine sampling. In addition, as seen in these two cases, the rhabdomyoblastic element could also lose their other epithelial characteristics such as cytokeratin reactivity. In such case, diagnosis of de novo rhabdomyosarcoma is basically a function of extensiveness of tissue sampling except for alveolar rhabdomyosarcoma for which molecular analysis for PAX3/7-FHKR translocation can be used to confirm the diagnosis. As illustrated in our cases, majority of the rhabdomyoblastic tumors of the bladder are likely rhabdomyosarcomatous carcinoma as small foci of carcinomatous element are only detectable through extensive sampling of the single or multiple specimens and pure rhabdomyoblastic tumor (a.k.a de novo rhabdomyosarcoma) is very rare and the diagnosis should be made with extreme caution.

The other challenge is differentiating rhabdomyosarcoma and rhabdomyoblastic carcinoma from small cell carcinoma. As some previous studies showed [7], we also find that there is significant overlapping of morphologic features between small cell carcinoma, rhabdomyosarcoma and carcinoma with extensive rhabdomyoblastic differentiation in urinary bladder. Small cell carcinoma of bladder is a rare tumor with incidence reported between 0.5% and 1% of the bladder cancers in retrospective studies [18-20]. It can coexist with urothelial carcinoma. It has been shown that similar patterns of allelic loss in the small cell carcinoma component coexisting with urothelial carcinoma. The similar patterns of allelic loss leads to the hypothesis that these cells have common clonal origin [21]. The prognosis for small cell carcinoma of the urinary bladder is poor, with overall 1-year and 5-year disease-specific survival rates of 56% and 16% respectively [19]. The treatment for bladder small cell carcinoma is not standardized. However, chemotherapy played a prominent role in the management of these tumors [22]. Recent development includes the use of adjuvant platinum-based chemotherapy for advanced disease. For adult bladder RMS, treatments are variable, including surgical resection, radiotherapy, chemotherapy or combined therapy. Prognostic markers with a worse prognosis included nonembryonal histology, tumor invasion and tumor size > 5 cm in children' RMS [23]. Management of carcinoma with extensive rhabdomyosarcomatous differentiation is not well defined. The preferred treatment appears to be cystectomy followed by radiation therapy or chemotherapy [24].

Due to the difference in management, proper classification of the rhabdomyoblastic tumors in bladder and differentiate these tumors from morphologic mimics such as small cell carcinoma are important. In this study, we compared the expression of cytokeratin, myogenin, synaptophysin and chromogranin in rhabdomyosarcomatous tumor and small cell carcinoma of the bladder. As shown in Table 4, areas of rhabdomyosarcomatous differentiation were positive for desmin and myogenin, negative for cytokeratin and chromogranin in all cases. In this study, we reported strong positivity for synaptophysin detected in the RMS component of one of the sarcomatoid carcinomas, which was not reported previously. As reported [7], positivity for synaptophysin can also be detected in pure RMS, and hence has little utility to differentiate rhabdomyosarcomatous tumors from other mimics with neuroendocrine differentiation such as small cell carcinoma. In contrast, small cell carcinoma components, whether it is pure or admixed with urothelial carcinoma, were positive for cytokeratin and largely positive for chromogranin, and negative for myogenin. FISH analysis was performed on paraffin-embedded formalin-fixed tissue in three cases of rhabdomyosarcomatous tumor of the bladder tumors to detect rearrangement of the FKHR (13q14) region which is hallmark of alveolar rhabdomyosarcoma. No FKHR rearrangement was detected in any of these three cases. The negativity for such arrangements does not support the diagnosis of alveolar type of rhabdomyosarcoma. Primary de novo rhabdomyosarcoma of any type is very rare in adult bladder. In this setting, only alveolar rhabdomyosarcoma can be reliably diagnosed by molecular test for PAX3 or 7-FKHR translocation. If the tumor is negative for PAX3 or 7-FKHR translocation and lack of morphologic pleomorphism, most of rhabdomyosarcomatous tumors arising from adult bladder are likely of rhabdomyosarcomatous carcinoma, a form of sarcomatoid carcinoma. Embryonal rhabdomyosarcomas can arise in bladder wall in pediatric patient but are extremely rare in adult patient. Clear morphologic differential separation of de novo embryonal rhabdomyosarcoma from those rhabdomyosarcomatous carcinomas would be a challenge and depend upon one's will to search for evidence of carcinoma in specimen. Unless a molecular test reveals evidence of alveolar rhabdomyosarcoma, tumors with extensive rhabdomyosarcomatous differentiation of the adult bladder should be either classified as rhabdomyosarcomatous sarcomatoid carcinoma when epithelial differentiation is identified or rhabdomyosarcomatous tumor of the bladder when no epithelial differentiation can be detected after reasonable effort to search such evidence in a caveat that there is no standard for what is considered reasonable effort in evaluating such specimen. Such diagnostic approach acknowledges the difficulty in making morphologic differentiation between rare occurring de novo soft tissue rhabdomyosarcoma and rhabdomyosarcomatous sarcomatoid carcinoma in adult patients.

In summary, pure RMS is very rare in adult urinary bladder while rhabdomyosarcomatous sarcomatoid carcinoma is more common. Adequate sampling, search for admixed in-situ or invasive urothelial carcinoma and use of cytokeratin stain may help to reach a correct diagnosis. A descriptive diagnosis of rhabdomyosarcomatous tumor of the bladder is preferred in difficult cases. Rhabdomyosarcoma and rhabdomyosarcomatous sarcomatoid carcinoma can be differentiated from small cell carcinoma of the bladder by immunohistochemical evaluation of cytokeratin and chromogranin and myogenin.

## Competing interests

The authors declare that they have no competing interests.

## Authors' contributions

ZB and PJZ conceived, designed and carried out the project. All authors read and approved the final manuscript.
